# Gene expression analysis indicates reduced memory and cognitive functions in the hippocampus and increase in synaptic reorganization in the frontal cortex 3 weeks after MDMA administration in Dark Agouti rats

**DOI:** 10.1186/s12864-018-4929-x

**Published:** 2018-08-02

**Authors:** Peter Petschner, Viola Tamasi, Csaba Adori, Eszter Kirilly, Romeo D. Ando, Laszlo Tothfalusi, Gyorgy Bagdy

**Affiliations:** 1 Department of Pharmacodynamics, Semmelweis University, Nagyvarad ter 4., Budapest, H-1089 Hungary; 2 MTA-SE Neuropsychopharmacology & Neurochemistry Research Group, Nagyvarad ter 4., Budapest, H-1089 Hungary; 3 Department of Genetics, Cell- and Immunobiology, Semmelweis University, Nagyvarad ter 4., Budapest, H-1089 Hungary; 4 4 Retzius Laboratory, Department of Neuroscience, Karolinska Institutet, Retzius väg 8, 17177, Stockholm, Sweden; 5 NAP-2-SE New Antidepressant Target Research Group, Semmelweis University, Nagyvarad ter 4., Budapest, H-1089 Hungary

**Keywords:** Ecstasy, Endocannabinoid, CB1, RhoGTPase, Serotonin, Gene expression, Microarray, Eph receptors, CaMKII, NMDA2B

## Abstract

**Background:**

3,4-methylenedioxymethamphetamine (MDMA, “ecstasy”) is a widely used entactogenic drug known to impair cognitive functions on the long-run. Both hippocampal and frontal cortical regions have well established roles in behavior, memory formation and other cognitive tasks and damage of these regions is associated with altered behavior and cognitive functions frequently described in otherwise healthy MDMA users. Meanwhile, in post-traumatic stress disorder (PTSD) patients seem to benefit from therapeutic application of the drug, where damage in hippocampal cue extinction may play a role. The aim of this study was to examine the hippocampus, frontal cortex and dorsal raphe of Dark Agouti rats with gene expression arrays (Illumina RatRef bead arrays) looking for possible mechanisms and new candidates contributing to the consequences of a single dose of MDMA (15 mg/kg) 3 weeks earlier.

**Results:**

The number of differentially expressed genes in the hippocampus, frontal cortex and the dorsal raphe were 481, 155, and 15, respectively. Gene set enrichment analysis of the microarray data revealed reduced expression of ‘memory’ and ‘cognition’, ‘dendrite development’ and ‘regulation of synaptic plasticity’ gene sets in the hippocampus, parallel to the downregulation of CaMK II subunits, glutamate-, CB1 cannabinoid- and EphA4, EphA5, EphA6 receptors. Downregulated gene sets in the frontal cortex were related to protein synthesis, chromatin organization, transmembrane transport processes, while ‘dendrite development’, ‘regulation of synaptic plasticity’ and ‘positive regulation of synapse assembly’ gene sets were upregulated besides elevated levels of a CaMK II subunit and NMDA2B glutamate receptor. Changes in the dorsal raphe region were mild and in most cases not significant.

**Conclusion:**

The present data raise the possibility of new synapse formation / synaptic reorganization in the frontal cortex 3 weeks after a single neurotoxic dose of MDMA. In contrast, a prolonged depression of new neurite formation in the hippocampus is proposed by downregulations of members in long-term potentiation pathway and synaptic plasticity emphasizing the particular vulnerability of this brain region and proposing a mechanism responsible for cognitive problems in healthy individuals. At the same time, these results underpin benefits of MDMA in PTSD, where the drug may help memory extinction.

**Electronic supplementary material:**

The online version of this article (10.1186/s12864-018-4929-x) contains supplementary material, which is available to authorized users.

## Background

Ecstasy (3,4-methylenedioxymethamphetamine, MDMA) is an amphetamine derivative widely abused for its euphoric and prosocial (entactogenic) effects in developed countries [1, 2]. The acute indirect monoaminergic agonist effects of MDMA are mainly mediated by an increase in serotonergic, noradrenergic and dopaminergic neurotransmission of the brain by reversing transmembrane transporter functions, which are normally responsible for the uptake of neurotransmitters from the synaptic cleft [1–3]. However, in the long-run, a decrease in serotonergic markers was reported in experimental animals and also in human users suggesting a long-term selective vulnerability of the serotonergic system [2, 4–6]. Functional deficits could also be observed in humans and in rodents, e.g. impaired decision making, sleep disturbances, increased anxiety and impulsivity levels, elevated aggression, learning and memory impairments and depression [2, 5, 7–10]. In addition to the selective damage observed in serotonergic neurons, MDMA may also cause more wide-spread changes, like production of toxic metabolites and free radicals or the disruption of local cerebral blood flow and glucose utilization, which might cause alterations in the nutrition-supply of neurons [4, 11–14].

The serotonergic projections in the mammalian brain, the primary targets of MDMA’s effects in rats, originate from the raphe nuclei in the brainstem. Dorsal (DR) and median raphe nuclei innervate upper brain structures, including the frontal cortical regions and the hippocampus (HC) [15–18]. The frontal cortex (FC) plays major roles in risk evaluation, executive functioning, and working memory, while its malfunctions may be associated with neuropsychiatric diseases [19–22]. At the same time, HC has a pivotal role in contextual and hereby spatial memory formation [18, 23], thus, all of the latter regions are candidates for long-run functional consequences caused by MDMA.

Despite the observed serotonergic deficits, recent criticism of these studies raised the possibility of the therapeutic application of MDMA. The Multidisciplinary Association for Psychedelic Studies (www.maps.org) is involved in studies investigating drug-assisted psychotherapy with MDMA in post-traumatic stress disorder (PTSD) patients. As a result, Mithoefer et al. demonstrated an 83% clinical response rate to psychotherapy with tolerable side effects in the MDMA group compared with 21% in the control group [24, 25]. However, mechanisms responsible for these promising results in severe cases of PTSD remain to be elucidated in the different brain regions that are implicated in cognition and behavioral alterations.

Parallel to neuronal damage neuroprotective mechanisms can occur and, later in time, recovery processes also may begin. Heat-shock proteins (HSPs) can ameliorate the damage caused by cellular stress of different origin e.g. hyperthermia, ischemia, or excessive production of free radicals [26]. Elevated levels of HSP27 in the FC and HC 3 days after MDMA treatment was demonstrated by Adori et al. and this elevation persisted until at least 7 days in the HC but normalized in the FC by this time [4]. Brain-derived neurotrophic factor (BDNF), a well-characterized member of neurotrophic factors, is involved in several processes maintaining central nervous system (CNS) functions like dendritic arborization, synaptogenesis and activity-dependent potentiation (for review see [27]). A study elucidating MDMA’s effects on BDNF mRNA expression reported ever increasing elevations in FC up to 7 days after MDMA administration while in the HC a decrease was evident [28]. Investigation of MDMA’s long-term effects revealed that in the parietal cortex BDNF protein levels peaked at 8 weeks after an initial decline but in the HC no significant change could be reported [29]. Differences were also demonstrated on the long-run between HC and temporal cortex in tryptophan hydroxylase levels, the 5-HT synthesis enzyme, following binge or single-dose administration of MDMA [30]. All of the latter results suggest different recovery capacities of the HC and FC, but the detailed biochemical mechanisms responsible for these differences remained so far less investigated at later time points. We speculated that these consequences might be already visible at 21 days following a single dose of MDMA thus we performed our analysis 3 weeks after drug administration to investigate both recovery processes and downstream mediators of already mentioned functional effects at this time point [5, 7].

Studies examining transcriptional changes following MDMA administration are scarce, only few reports evaluated alterations in mRNA levels of genes which were assumed to be related to MDMA effects (see e.g. [5, 28, 31–33]).

Thus, the aim of this study was to identify downstream transcriptional consequences of MDMA (which may be related to functional alterations of the drug) and to find possible new targets of regulatory mechanisms by using large-scale gene expression profiling in the HC, FC, and DR regions of Dark Agouti (DA) rats 21 days after a single-dose MDMA administration. Additionally, we also addressed whether signs of functional recovery on the molecular level can occur in the FC and HC and if so, whether they differ in quality or quantity in these two regions. In order to achieve our goals we used a dosage regimen of 15 mg/kg in the DA rat strain, which was utilized for the following reasons: 1) it is a commonly used strain in MDMA research, 2) DA rats are more similar to each other compared to other rat strains, thus, on a genome-wide scale our results remain more comparable with other experiments, 3) we were able to compare current results with those from our previous experiments with the same strain and same dosage regimen.

## Results

### General overview of gene expression alterations

Comparison of the gene expression profiles showed 615 differentially expressed genes in the MDMA treated group compared to the saline control (minimum probability of positive log ratio [MinPplr] < 0.001). From 155 significant genes in the FC region 66 were up- and 89 downregulated. In the HC region 481 genes showed altered expression, 171 and 310 genes were up- or downregulated, respectively. Only 14 unique genes were altered in the DR region, 11 showed elevated expression and the remaining 3 showed a decrease compared to the control group. All genes emphasized in the chapters below were selected by individual considerations of MDMA’s known effects and related literature data. For full results see Additional file 1: Table S1.

The gene set enrichment analysis (GSEA) revealed 55, 18 and 1 differentially regulated gene sets in the FC, HC and DR regions, respectively.

### Frontal cortex

#### Differentially expressed genes

MDMA caused a significant overexpression of long-term potentiation (LTP) pathway genes (*Camk2g* and *Camk1g*) and the ionotropic glutamate receptor, NMDA2B (*Grin2b*). The alpha subunit of the heat shock protein 1 (*Hspca,* HSP90α) and heat shock factor 2 (*Hsf2*) were downregulated, similarly to high-affinity glial glutamate transporter (*Slc1a3*) (See Table 1 and Additional file 1: Table S1 for all significantly altered genes).

**Table 1:** Selection of significantly altered genes by single-dose MDMA treatment in the different brain regions

		MinPplr	Fold Change (on log2 scale)
Frontal Cortex			
*Camk2g*	Calcium/calmodulin-dependent protein kinase II gamma	0.000127	0.900
*Camk1g*	Calcium/calmodulin-dependent protein kinase I gamma	0.000174	0.383
*Grin2b*	Glutamate receptor, ionotropic, NMDA2B (Grin2b)	0.000395	0.448
*Hspca*	Heat shock protein 90α	0.000677	−0.398
*Slc1a3*	Solute carrier family 1 (glial high affinity glutamate transporter), member 3	0.000417	−0.724
Hippocampus			
*Gria3*	Glutamate receptor, ionotropic, AMPA3	5.23E-05	−0.916
*Grin2a*	Glutamate receptor, ionotropic, N-methyl D-aspartate 2A	5.42E-08	−1.610
*Camk2g*	Calcium/calmodulin-dependent protein kinase II gamma	1.46E-08	−1.451
*Camk2b*	Calcium/calmodulin-dependent protein kinase II beta	1.02E-08	−1.751
*Kalrn*	Kalirin, RhoGEF kinase	2.69E-05	−0.419
*LOC316539*	Eph receptor A4	1.82E-05	−0.729
*Epha5*	Eph receptor A5	3.29E-09	−1.065
*Epha6*	Eph receptor A6	7.39E-05	−0.484
*Camk2n2*	Calcium/calmodulin-dependent protein kinase II inhibitor 2	3.44E-06	0.345
*Gabre*	Gamma-aminobutyric acid A receptor, epsilon	0.000525	0.268
*Atp2b3*	ATPase, Ca++ transporting, plasma membrane 3	2.41E-12	−0.567
*Atp2b1*	ATPase, Ca++ transporting, plasma membrane 1	1.16E-05	−0.863
*Slc5a3*	Solute carrier family 5 (inositol transporters), member 3	9.94E-10	−1.356
*Kcnd2*	Potassium voltage gated channel, Shal-related family, member 2	7.56E-07	−1.476
*Kcnc2*	Potassium voltage gated channel, Shaw-related subfamily, member 2	6.11E-07	−0.905
*Cnr1*	Cannabinoid receptor 1	3.1E-06	−0.793
Dorsal Raphe			
*Slc6a5*	Solute carrier family 6 (neurotransmitter transporter, glycine), member 5	0.000153	1.143
*Hsd11b1*	Hydroxysteroid 11-beta dehydrogenase 1	1.19E-08	0.450
*Dao1*	D-amino acid oxidase	0.000384	0.451

This table summarizes the genes significantly (minimum probability of positive log ratio (MinPplr) < 0.001) altered in the frontal cortex (FC), hippocampus (HC) and dorsal raphe (DR) of Dark Agouti (DA) rats after a single-dose 3,4-methylenedioxymethamphetamine (MDMA) treatment (15 mg/kg, intraperitoneal) 3 weeks earlier. The genes were selected based on literature data and individual considerations, for all the significantly changed genes see Additional file 1: Table S1. See text for further details

#### Gene set enrichment analysis

Altogether 55 gene sets were differentially enriched in the FC after the single-dose MDMA treatment, containing both our literature-based, individually chosen (Additional file 2: Table S2) and Msig DB C5 gene sets. Additionally, we used GSEA network analysis on significantly enriched GO terms to determine biologically relevant processes shown in Table 2.

**Table 2:** Biologically Relevant Processes with Enriched Gene Sets in the Frontal Cortical Region

Biological Process	Number of Enriched Gene Sets Related to the Process	Direction
Protein synthesis and localization	21	⬇
Transmembrane transport	10	⬇
Nucleocytoplasmic transport	7	⬇
Cell growth	6	⬆⬇
Others	4	⬆⬇
Chromatin maintenance	3	⬇
Dendrite and synapse development	3	⬆
Oxidoreductase activity	2	⬇

This table summarizes the biological processes significantly altered in the frontal cortex (FC) of Dark Agouti (DA) rats after a single-dose 3,4-methylenedioxymethamphetamine (MDMA) treatment (15 mg/kg, intraperitoneal) 3 weeks earlier. The processes were created based on the network analysis of gene set enrichment x analysis and individual considerations. All significantly enriched GO terms are included in one of the processes. Downward arrows represent down-, upward arrows represent upregulations. In some of the biological processes both up- and downregulated gene sets were present (mixed up- and downward arrows). See text for further details

The biological process with the most prominent alterations contained 21 significantly dysregulated gene sets related to protein synthesis and protein localization within the neurons, and 10 significantly enriched gene sets were involved in transport processes. All of these 31 gene sets were, without exception, downregulated in the FC region, together with gene sets responsible for chromatin maintenance. The dendrite and synapse development gene sets were upregulated 3 weeks after MDMA administration (Table 2, in all cases, *p* < 0.05, and FDR < 0.25, see Additional file 3: Table S3 for detailed and full results).

**Fig. 1:**
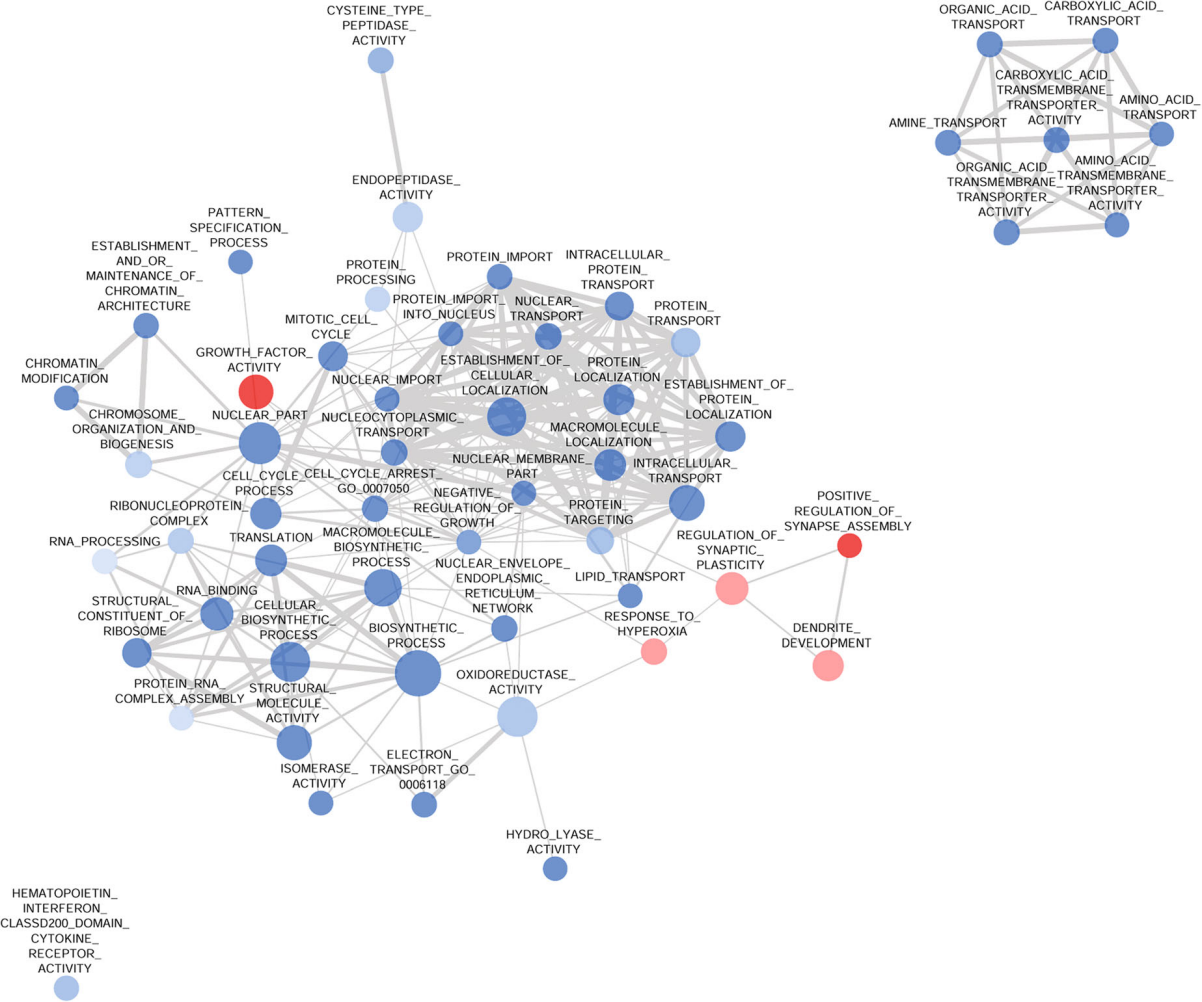
Network Analysis of the GSEA Results in the Frontal Cortex. The network shows significantly (nominal *p* < 0.05, false discovery rate < 0.25) enriched gene ontology (GO) terms in the gene set enrichment (GSEA) analysis in the frontal cortex (FC) of Dark Agouti rats 3 weeks after a single-dose (15 mg/kg, intraperitoneal) 3,4-methylenedioxymethamphetamine (MDMA) administration. Blue and red circles represent down- and upregulation of the associated GO terms, respectively. The size of the nodes is proportional with the number of genes in the GO term and the thickness of the grey edges represents the number of common genes between two GO terms, if any. Most of the processes in the FC were downregulated. The cellular biosynthetic process and establishment of cellular localization GO terms are in central positions of two different groups while chromosome and chromatin organization, and transmembrane transport processes form the third and fourth group of tightly related, downregulated gene sets. These alterations suggest a general neurotoxic effect while the upregulation of gene sets related to synapse formation raises the possibility of a parallel running recovery process. The expression of the only immunologically related GO term was attenuated. See text for further details

We also used in silico GSEA network analysis (Fig. 1) on significantly enriched gene ontology (GO) terms, which represents functional connectivity and was applied to determine biologically relevant processes shown in Table 2.

### Hippocampus

#### Differentially expressed genes

Changes in major hippocampal neuroplasticity related pathways included reductions in glutamatergic GRIN2A (*Grin2a)* and AMPA3 (*Gria3*) mRNA levels, parallel with downregulations in a variety of LTP pathway members, like calcium/calmodulin dependent protein kinase (CaMK) II genes (*Camk2g, Camk2b)*, kalirin*, (Kalrn*) and EphA4 (*LOC316539)*, *Epha5* and *Epha6* receptors, members of ephrin signaling. Accordingly, an inhibitor of the CaMK II (*Camk2n2*) was upregulated along with a GABA-A receptor subunit (*Gabre*). The mRNA levels of *Atp2b3*, *Atp2b1* calcium transporting ATP-ases and *Slc5a3*, an inositol transporter was also decreased. Some of the voltage-gated potassium transporter genes (*Kcnd2*, *Kcnc2*) were also downregulated and type 1 cannabinoid receptor (*Cnr1,* CB1) showed decreased levels in HC, too. (See Table 1 and Additional file 1: Table S1 for all significantly altered genes).

#### Gene set enrichment analysis

**Fig. 2:**
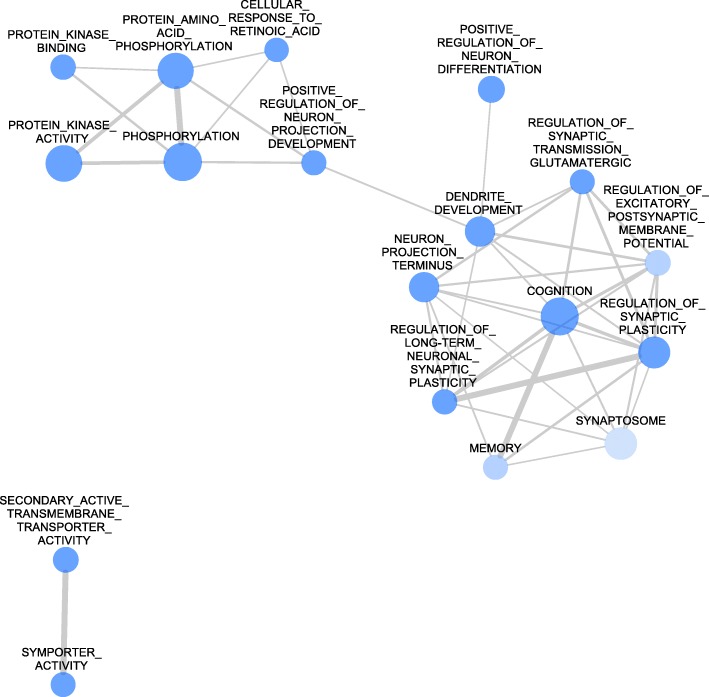
Network Analysis of the GSEA Results in the Hippocampus. The network shows significantly (nominal p < 0.05, false discovery rate < 0.25) enriched gene ontology (GO) terms in the gene set enrichment (GSEA) analysis in the hippocampus (HC) of Dark Agouti rats 3 weeks after a single-dose (15 mg/kg, intraperitoneal) 3,4-methylenedioxymethamphetamine (MDMA) administration. Blue circles represent the downregulation of the associated terms, while red circles would represent upregulations. The size of the nodes is proportional with the number of genes in the GO term and the thickness of the edges between lines represents the similarity coefficient. All processes in the HC are downregulated, and two well characterized groups of processes emerge. On one hand the processes related to cognition and memory, e.g. regulation of synaptic plasticity, dendrite development and regulation of glutamatergic synaptic transmission gene sets form a diverse group. On the other hand the processes related to kinase activity form another network. See text for further details

The GSEA analysis revealed altogether 18 differentially represented gene sets in the HC region, including both the Msig DB C5 gene set database and individually chosen gene sets based on the literature (see Additional file 2: Table S2). Two particularly important gene sets, ‘memory’ [GO:0007613] and ‘cognition’ [GO:0050890] were underexpressed in HC samples and multiple gene sets related to the molecular function of kinases were also downregulated. Other, negatively influenced processes included synaptic plasticity and dendrite / synapse development and the regulation of glutamatergic neurotransmission (Table 3, in all cases, *p* < 0.05, and false discovery rate (FDR) < 0.25, see Additional file 3: Table S3 for detailed and full results). Figure 2 summarizes the main findings of the gene set analyses in a network form based on the GSEA network analysis.

**Table 3:** Biologically Relevant Processes with Enriched Gene Sets in the Hippocampal Region

Biological Process	Number of Enriched Gene Sets Related to the Term	Direction
Protein Phosphorylation	4	⬇
Dendrite and synapse development	4	⬇
Synaptic plasticity	4	⬇
Transmembrane transport	2	⬇
Others	2	⬇
Memory, cognition	2	⬇

This table summarizes the biological processes significantly altered in the hippocampus of Dark Agouti rats after a single-dose 3,4-methylenedioxymethamphetamine (MDMA) treatment (15 mg/kg, intraperitoneal) 3 weeks earlier. The processes were created based on the network analysis of gene set enrichment analysis and individual considerations. All significantly enriched GO terms are included in one of the processes. Downward arrows represent downregulations. See text for further details

### Dorsal raphe

#### Differentially expressed genes

In the DR region the glycine neurotransmitter transporter (*Slc6a5*), the D-amino acid oxydase (*Dao1*) and the 11-beta-hydroxisteroid dehydrogenase (*Hsd11b1,* 11β-HSD1) genes were upregulated among others (See Table 1 and Additional file 1: Table S1 for all significantly altered genes).

#### Gene set enrichment analysis

In the region only one gene set, namely ‘caspase activation’ [GO:0006919] was significantly downregulated after the single-dose MDMA treatment. No upregulated gene sets could be observed (in all cases p < 0.05, and FDR < 0.25). The full results of the GSEA analysis in the DR region are shown in Additional file 3: Table S3.

### Heatmap analysis

**Fig. 3:**
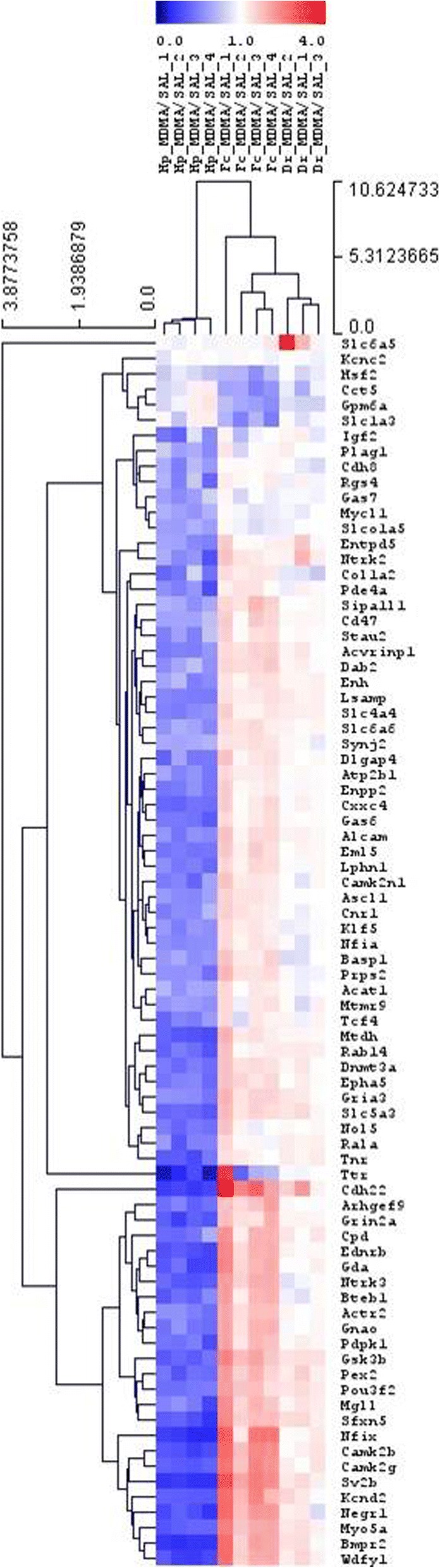
Two-way hierarchical clustering of representative genes selected from all three brain regions. Genes modulated more than 1.5 or less than 0.5 are clustered (Euclidean distance, average linkage) into the heatmap from all three brain regions (hippocampus [HC], frontal cortex [FC] and the dorsal raphe [DR]) of Dark Agouti rats 3 weeks after a single-dose of (15 mg/kg, intraperitoneal) MDMA. The brain regions are unequivocally separated by this analysis, suggesting markedly different effects of MDMA in the different regions. See text for further details

The heatmap (Fig. 3) shows genes after two-way hierarchical clustering comparing their expression levels among all three regions. It provides a different insight into the transcriptional changes after MDMA treatment. In the HC region nearly all of the genes were downregulated. In contrast, most of those genes that were downregulated in the HC were found to be upregulated in the FC, suggesting marked differences between the two regions. Changes in the DR were scant independently of alterations in the other two regions.

## Discussion

In this study we evaluated the transcriptional consequences 3 weeks after a single neurotoxic dose of MDMA in DA rats with gene expression arrays in three different brain areas. The FC region exhibits wide-scale negative changes in basic anabolic and transport processes and the upregulations of the ‘dendrite development’, ‘regulation of synaptic plasticity’ and ‘positive regulation of synapse assembly’ gene sets suggest a partial new synapse formation/synaptic reorganization in this region on the transcriptional level. MDMA’s effects indicate alterations in cognition and memory related processes with the possible involvement of the LTP pathway, CB1 and Eph receptors in the HC. These differences between the FC and HC point to markedly different transcriptional responses of these two brain regions 3 weeks after a single dose MDMA administration.

### Frontal cortex

While alterations in expression of 5-HT markers in cortical regions are well-defined, studies examining other effects of MDMA on gene expression are scarce. Thiriet et al. examined 1176, toxicology-related genes in adult Sprague-Dawley rats and followed expression patterns up to 7 days after a 20 mg/kg single-dose MDMA administration [33]. They found nerve growth factor alterations and suggested cytoskeletal reorganization while in another study, Fernandez-Castillo et al. emphasized neuroinflammatory responses in MDMA-effects 8 h after repeated-administration in adult mice [32]. Martinez-Turillas et al. investigated BDNF augmentations in the FC region of Wistar rats up to 7 days after drug administration [28]. In our present study we examined gene expression patterns longer time (3 weeks) after a single neurotoxic dose of MDMA in the DA rat strain and found no overlap with previous transcriptomic investigations probably because of the different time point examined. We report wide-scale downregulation of genes involved in chromatin organization, nucleocytoplasmic transport, ribosome-related functions, protein synthesis/folding and transmembrane transport processes in the FC region (Table 2). It seems reasonable that the observed changes are long-term consequences of the acute general neurotoxic processes, like toxic metabolite formation, hyperthermic effect or free radical production or impairment in the autoregulation of cerebral blood flow [4, 11–14, 34–38]. The latter is even further supported by the upregulation of the response to hyperoxia gene set.

Motor regions in the FC are targets of thalamical inputs and contribute to motor system functions [39]. Studies in DA rats with the same MDMA administration protocol like in the recent experiment indicated chronic changes in motor activity [40–42]. Additionally, Karageorgiou et al. reported alterations in right supplementary motor area activation in human MDMA users on fMRI recordings [43]. Our results might reflect molecular changes in the FC that may relate to these observations on a functional level.

As from another functional perspective FC and prefrontal cortical regions (PFC) are not only responsible for motor functions, but are also closely related to different cognitive tasks, e.g. working memory, goal-directed behavior, and executive functions in rats [20, 44–46]. In our experiment FC samples contained regions from primary and secondary (supportive) motor cortices principally and likely some parts of PFC [46]. Thus, inhibition of certain biosynthetic processes found in the present study might be distinctly involved in the cognitive decline of heavy MDMA users.

We have previously demonstrated that HSP90 staining is not influenced by MDMA either 3 days or 1 week after administration in DA rats with the same administration protocol [4]. The downregulation of *Hspca* gene mRNA encoding for HSP90α, therefore, is surprising and may indicate changes later in time that might be triggered by *Hsf2* (also downregulated in the present experiment) able to bind promoter regions of *Hspca* [47]. Nevertheless, further studies are definitely required to unravel the exact roles of these factors in the FC of DA rats after single-dose MDMA administration.

A possible recovery mechanism on a network level marked by the upregulation of LTP members, like CaMK subunits *Camk2g* and *Camk1g* and the ionotropic glutamate receptor, NMDA2B (*Grin2b*), is suggested. Activation of CaMK II is both necessary and sufficient for LTP induction under physiological circumstances and it remains one of the most important factors in this process and, hence, in cognition itself [48]. The preceding Ca-influx which normally activates CaMK II is triggered by activation of NMDA-type glutamate receptors and, while different NMDA subunits may regulate different behaviors, all are involved in LTP [48, 49]. Physiologically, after elevations in intracellular Ca^2+^ concentrations, which render it into active conformation, CaMK II phosphorylates multiple targets within the neurons on their serine/threonine residues and ultimately recruits AMPA receptors into postsynaptic dendritic spine membranes accompanied by morphological changes of these membranes through guanine nucleotide exchange factors that activate Rho GTPases (RhoGEFs) [48]. According to our recent understanding, through these steps synapses are markedly sensitized for glutamate-induced depolarization, which is, in essence, LTP and is considered as the basic mechanism of cognitive functions on neuronal levels. LTP, while mainly associated with HC has important roles also in motor regions, PFC, and neocortical regions and it influences skill learning and executive functions alike [50, 51]. Our results indicate that the frontal cortical region reacts differently to a previous, single-dose MDMA challenge than HC in DA rats (see in Hippocampus chapter). While individual cells may show impaired biosynthetic processes, these alterations seem to be compensated on a network level by the possible strengthening of neuronal connections, a mechanism which provides the possibility of functional restoration following injuries of the central nervous system [52]. These conclusions on the gene level are partially underpinned by the GSEA, since gene sets related to dendrite and synapse development were upregulated. While connectivity may be enhanced, indication for enhanced LTP is provided only on a gene level (elevated *Camk2g, Camk1g* and *Grin2b*), and we have to note that gene sets could not unequivocally confirm such processes.

**Fig. 4:**
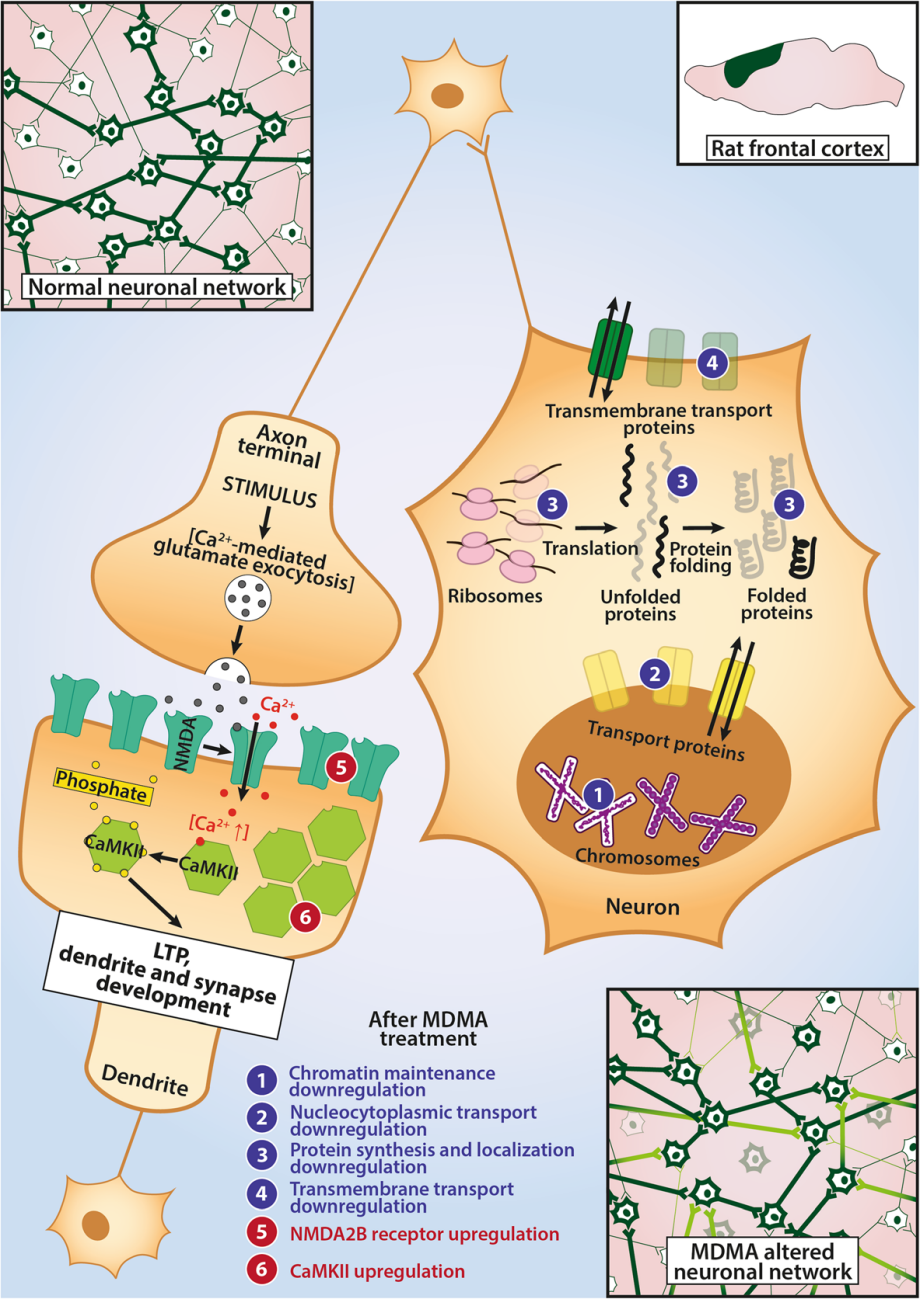
A Schematic Representation of MDMA’s Sites of Action in Frontal Cortex. This figure summarizes the effects of a single-dose (15 mg/kg, intraperitoneal) 3,4-methylenedioxymethamphetamine (MDMA) administration 3 weeks earlier on the frontal cortex (FC) of Dark Agouti rats and provides a hypothetical framework. On one hand, neurons of neuronal networks within this region seem to suffer wide-scale downregulations (marked with numbers in blue circles in the neuron on the right hand side) in their basic functions, like transport processes, chromatin maintenance and protein synthesis and localization, as a possible consequence of MDMA’s free radical producing and hyperthermic effects. Since the downregulated processes would be important mechanisms for neuronal survival, neuronal networks might suffer from the loss of basic functions of individual cells. As compensation on a network basis, upregulations of the long-term potentiation (LTP) genes (like *Camk2g* calcium/calmodulin dependent kinase subunit and NMDA-receptor subunit *Grin2b*, marked by numbers in red circles in the neuron on the left hand side) along with pathway level upregulations suggest a possible ongoing recovery (as shown in bottom right). Please, note, changes represent mRNA level up/downregulations, no protein levels were measured

In summary, the downregulation of almost 50 gene sets related to biosynthetic processes in the FC may reflect consequences of general neurotoxic effects not related to specific pathways (see Fig. 4). At the same time, the upregulation of the gene set responsible for dendrite development in this brain region may point to a starting recovery process/synaptic reorganization and could be a sign of a compensatory mechanism ameliorating MDMA’s acute effects 3 weeks after the administration in DA rats.

### Hippocampus

In the HC region we report downregulations in CaMK II subunits, *Camk2g* and *Camk2b* and upregulation of an inhibitor of CaMKs parallel with decreased level of a regulatory subunit of NMDA glutamate receptors, *Grin2a*. Moyano et al. observed attenuated levels of CaMK II after 1 week cessation following an MDMA treatment protocol upon a repeated administration, but could not demonstrate altered levels of CaMK II or NMDA-receptor subunits without the acute challenge, hence, concluded that MDMA cannot cause chronic alterations in CaMK II or NMDA-subunit levels in the HC [53]. We show that later, namely after 3 weeks, CaMK II and Grin2a show decreased mRNA levels. Chronic downregulations of CaMK II and an NMDA channel mRNAs may mark central alterations that can ultimately lead to losses in cognitive performance via the disruption of LTP. In the present study decreased expression of Ca-transporters, like *Atp2b3* and *Atp2b1*, further support the notion of altered calcium homeostasis that may affect CaMK II activation, while downstream effectors of the LTP pathway, AMPA3 and kalirin, a RhoGEF kinase, were also significantly downregulated. Taken together, besides CaMK II and *Grin2a*, mRNA levels of several components necessary for proper LTP are negatively influenced 3 weeks after single-dose MDMA administration in the used rat strain. Furthermore, results from GSEA support the gene level data showing downregulation of protein phosphorylation, memory, cognition, synaptic plasticity and synapse/dendrite development gene sets, correlates of the mRNA alterations on a pathway level (though LTP pathway directly remained non-significant, see Fig. 2 and Additional file 3: Table S3). Thereby, HC shows a contrast to FC, where some genes of the LTP pathway showed opposite changes. Such differences between the HC and FC suggest that different memory types with different relative involvement of these regions may show different responses to the drug. Indeed, a functional study investigating such differences following binge administration of MDMA, reported rats learning working-memory related tasks (mainly FC mediated) faster on the long-run compared to spatial reference memory (mainly HC mediated) in an 8-arm radial maze challenge [54]. In summary, these results indicate that the MDMA-caused lasting cognitive impairments in humans and experimental animals [2, 5, 8–10, 55, 56] may be partially consequences of transcriptional downregulations in essential elements of the CaMK II-mediated pathway in the HC at a chronic time point after use.

In this region we also show decreased mRNA levels of EphA4, EphA5 and EphA6 receptors. These membrane-anchored receptors only exert their actions upon direct cell-cell contact and, after binding, both the receptor and the similarly membrane-anchored ligand start intracellular signaling processes [57]. Eph receptors are suggested to be involved in the development and maintenance of HC and its cognitive functions through modulating synapse formation and glutamatergic LTP [58]. Simón et al. demonstrated that reductions in HC EphA4 levels preceded the development of attenuated object recognition and spatial memory in a mouse model of Alzheimer-disease [59, 60], while *Epha6* KO mice showed impaired memory using a fear conditioning training paradigm [61]. Although in comparison with EphA4 and EphA6, EphA5 is weakly labelled under physiological conditions in the HC, it may also be necessary for proper neuronal projections in the region [62].

Another gene downregulated in the current setup was the CB1 receptor. Nawata et al. also investigated CB1 receptor mRNA levels in the HC regions of mice up to 7 days following the cessation from repeated MDMA administration and they reported an increase 7 days, but not 1 day after last treatment [56]. CB1 receptors are important components in suppression of excitatory impulses in the HC through inhibition of presynaptic neurotransmitter release [63]. It is accepted that suppression of synaptic glutamate levels in the HC by cannabinoid agonists cause impairments in cognitive functions and, accordingly, antagonists may improve them [64]. Indeed, Nawata et al. also showed that a cannabinoid antagonist was able to ameliorate the MDMA induced cognitive impairments at 7 days after withdrawal from the drug suggesting that elevated CB1 levels may be important in this effect [56]. Here we report downregulations, which suggest alternating temporal patterns or species differences of this receptor after MDMA use and, hence, application of CB1 antagonists at later time points may have different consequences, at least in DA rats. Nevertheless, increased intracellular Ca^2+^ levels, a result of NMDA channel activation could directly induce endocannabinoid release in HC neurons and, thus, one may speculate that decreased Ca^2+^ levels (as a consequence of *Grin2a* downregulation) might be the sources for downregulations of CB1 receptors observed in the current paradigm [65].

Potassium channels play important roles in the maintenance of intracellular ion concentrations, the excitability of the neurons and in LTP, where long-term adaptations in excitability occur. The *Kcnd2* gene encodes for a Shal-related potassium channel, Kv4.2, which is responsible for A-type K^+^ currents in hippocampal pyramidal neurons [66]. Varga et al. demonstrated that CaMK II activation induces elevations in functional cellular Kv4.2 proteins, thus, the downregulations of Kv4.2 mRNA in our study might reflect the attenuation in CaMK II functions discussed previously [67], along with decreased *Kcnc2* levels encoding the Kv3.1b potassium channel [68].

As we have only investigated one dose of MDMA and only at 3-weeks after administration, our results are less generalizable. However, we would like to point to a possible connection between our results on a transcriptomic level and the recent successful clinical application of the drug in combination with psychotherapy in PTSD patients [25]. While in otherwise healthy human individuals the alterations discussed above can be detrimental on cognitive functions, in disease, loss of negative memory clues may be beneficial [69]. In PTSD it has been long proposed that dysfunctions in memory extinction may be involved in the maintenance of the symptomatology [70]. If our results can be extrapolated on an interspecies scale and onto the protein level by subsequent confirmatory analyses, they may represent molecular mechanisms through which MDMA may induce extinction of negative clues in HC circuitries and could benefit PTSD patients.

**Fig. 5:**
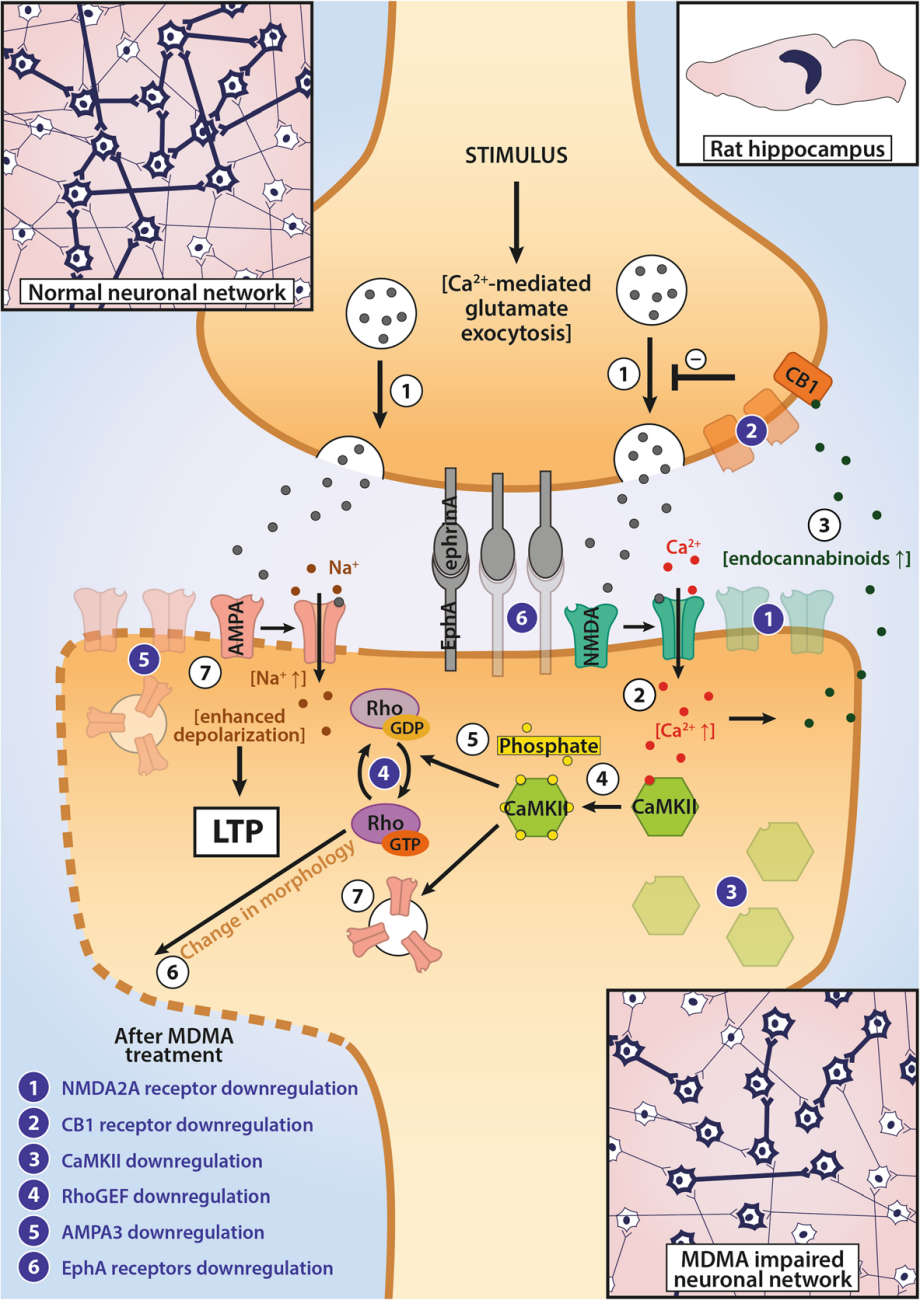
A Schematic Representation of MDMA’s Sites of Action in Hippocampus. This figure summarizes the effects of a single-dose (15 mg/kg, intraperitoneal) of 3,4-methylenedioxymethamphetamine (MDMA) 3 weeks earlier on hippocampal neurons in Dark Agouti rats. White circles represent molecular events explained in this legend. Based on current knowledge, neuronal long-term potentiation (LTP) would be initiated by glutamate release from synaptic vesicles (1) and their binding to NMDA-type glutamate receptors, which after activated, let Ca2+ flow into the cells (2). The elevated intracellular Ca-levels, on one hand, cause endocannabinoid synthesis acting as a negative feedback mechanism via cannabinoid 1 (CB1) receptors (3), while on the other hand, activate calcium/calmodulin dependent kinase II (CaMK II) and induce autoactivation of the enzyme (4). The active CaMK II molecules phosphorylate intracellular targets, thereby activating Rho GTPases (5) to induce changes in synaptic membrane morphology (6) and, thereby, let AMPA glutamate receptors be expressed in postsynaptic membranes (7). The newly recruited AMPA receptors may react to presynaptic glutamate release causing elevated excitability upon repeated stimuli and a strengthening of synaptic transmission. Downregulations caused by the single-dose administration are marked by blue numbers and show that besides of decreased mRNA levels found in our study in the subunits of the LTP-members (which was supported by downregulations in regulation of synaptic plasticity, cognition and memory gene sets), the drug also induced downregulation in Eph receptors, which may be responsible for direct cell-cell contacts in synapses. These changes indicate that the connectivity and thus, hippocampal neuronal network functions might be damaged as a consequence of the administration of the drug. Please, note, changes represent mRNA level up/downregulations, no protein levels were measured. See text for further details

Taken together, MDMA, 3 weeks after its use in a single dose, caused decreased mRNA levels of major components of the LTP pathway, ephrin signaling and CB1 receptors accompanied by downregulations in memory, cognition, synaptic plasticity and synapse/dendrite development gene sets in HC of DA rats (Fig. 5). Our results indicate that a central region of cognitive functioning, the HC, may be particularly vulnerable for MDMA’s toxic effects in DA rats.

### Dorsal raphe

The changes in the DR region were mild in line with our previous results suggesting that MDMA-caused damage to these neurons are restricted to serotonergic axon terminals instead of neuronal cell bodies directly [71]. The caspase activation gene set significantly changed in the present study was not supported by individual genes, or other gene sets related to apoptotic processes. The upregulation in 11β-HSD1 mRNA level might suggest a possible role of the hypothalamic-pituitary-adrenal axis and glucocorticoids in MDMA’s long-term consequences, an effect also proposed by others [72, 73]. 11β-HSD1 is responsible for the conversion of glucocorticoids into active form. Chronically elevated glucocorticoids were shown to be involved in aging and cognitive decline and it has been shown that 11β-HSD1 may induce such processes [74]. Since its expression was confirmed in the brainstem [75], 11β-HSD1 may be an interesting topic for further research in the region. Otherwise, the DR region seems to be mostly unaffected 3 weeks after a single-dose of MDMA administration in DA rats.

### Limitations

In the present study we have not elucidated the temporal patterns of the mRNAs. Further studies are needed addressing the time course of described alterations to elicit causative relations of these transcriptional processes in detail, especially, since some alterations in HC seem to be highly time-dependent and may influence therapeutic interventions of MDMA-induced cognitive changes.

We could not confirm the decreased expression of serotonergic markers in the present study. Both serotonin transporter and tryptophan hydroxylase mRNA levels were unaltered in the treatment group, which is in conflict with previous results: well established prolonged serotonergic depletion and decreased expression of serotonergic markers in both protein and mRNA levels after MDMA-treatment was demonstrated by our group earlier [4, 5]. Here we can assume that collection of DR samples was not precise enough and as we did not apply laser capture microdissection in this case, significant amount of surrounding tissue was perhaps cut out together with the DR and it may result a bias in the measurement of serotonergic markers. Notably, the decrease of serotonin transporter expression, measured by quantitative in situ hybridization, was approximately 20% in the same animal model 3 weeks after the MDMA treatment, compared to the control level, and this moderate alteration was significant only in case of the fine measurement of grain densities of individual cells but not with the measuring of the autoradiography signal on film [5].

On the other hand, microarray method has well-known drawbacks, when compared to polymerase chain reaction (PCR) methods or in-situ hybridization used in our earlier papers. Namely, limited amount of probes on the microarray may result in smaller fold change values. In addition, shorter oligomers used can result in more mismatch hybridization, which can overcome smaller changes in gene expressions, like that in the case of serotonin transporter. However, for this very reason we assume that the results presented here, with our significance criterion, are robust enough to overcome this bias.

We did not find alterations in the *Bdnf* gene expression, which is in agreement with our previous study where we demonstrated that (after a slight transient acute decrease) BDNF protein level was increased only 8 weeks after same MDMA dosage regimen in same rat strain [29].

Though members of LTP pathway were downregulated, we could not demonstrate a downregulation in the pathway itself by GSEA in HC. GSEA examines all of the genes of a certain pathway in the ordered list of genes (based on t-statistics) between the experimental groups. Significance of complete pathways therefore depends on all of the genes annotated to a given set. This could mean that other members, except those which were significant, in the LTP pathway remained mostly unchanged. However, on one hand, our present knowledge about CaMK II indicates that it can be solely responsible for the induction of LTP [48] and, on the other, related pathways (e.g. cognition and memory sets) were significant, therefore, we think this discrepancy is less of an importance.

We must also note the major limitation of transcriptomic studies, namely, mRNA levels do not necessarily reflect to the corresponding protein levels. At the same time, we have to point out that recent studies demonstrated mRNA levels as important regulators of protein levels [76, 77].

Finally, we also have to note that strain selection could have influenced the results because DA rats might be more prone to autoimmune neuroinflammation and glial activation [78], can have altered reactivity in the dopaminergic (and thus reward) system [79], may show elevated serotonergic neurotoxicity after MDMA [80] and DA rats are considered poor or intermediate metabolizers at CYP2D1 corresponding the human CYP2D6 that is implicated in MDMA metabolism [80]. While these effects may have influenced our observations, there is currently no direct proof that our findings would be substantially altered and conclusions limited by using this strain.

## Conclusion

We performed a genome-wide evaluation of transcriptional changes 3 weeks after a single-dose of MDMA in DA rats. The downregulated pathways in the FC were related to the basic mechanisms of the cell functionality in the absence of specific markers of certain pathways. Upregulation of ‘dendrite development’, ‘regulation of synaptic plasticity’ and ‘positive regulation of synapse assembly’ gene sets raise the possibility of new synapse formation/synaptic reorganization mechanisms in the region. All of these results point out to a starting reinstatement of the neuronal pathways and connections in the FC 3 weeks after a 15 mg/kg dose of MDMA. The HC region showed markedly different changes. Our data highlight decreased CaMK II, glutamatergic, Eph receptor and CB1 mRNA levels as potential downstream mediators of MDMA in the HC. In addition, GSEA showed downregulated ‘cognition’ and ‘memory’ gene sets similarly indicating decreased functionality of LTP and glutamatergic pathways. These results provide further molecular biological information on the transcriptional level, which may underpin the well-known cognitive deficits following MDMA use in humans and animals as well as might indicate a possible mechanism by which the drug can help extinction of negative memory clues in PTSD patients.

## Methods

### Animals

Altogether 21 male DA rats (Harlan, Olac Ltd., Shaw’s Farm, Blackthorn, Bicester, Oxon, UK) aged approximately 8 weeks (weighing 152 ± 3,58 g (SEM) at the beginning of the experiment) were used. The animals (four per cage) were kept under controlled environmental conditions along the whole experiment (temperature 21 ± 1 °C, humidity: 40–50%, 12 h light-dark cycle starting at 6:00 a.m.) and food and water were available for them ad libitum.

### Drug administration and experimental design

(±)3,4-methylenedioxymethamphetamine (Sanofi-Synthelabo-Chinoin, Hungary, purity > 99.5%) was dissolved in 0.9% NaCl (SAL) at an equivalent dose of 15 mg/kg free base and was administered intraperitoneally (i.p.) in a volume of 1 ml/kg. The dose of MDMA was selected based on the interspecies scaling ${\mathrm{D}}_{\mathrm{human}}={\mathrm{D}}_{\mathrm{animal}}{\left(\frac{{\mathrm{W}}_{\mathrm{human}}}{{\mathrm{W}}_{\mathrm{animal}}}\right)}^{0.7}$, where D are doses in mg and W are weights in kg [81]. By using W_animal_ = 0.152 kg, D_animal_ = 15 mg/kg * 0.152 kg, and W_human_ = 70 kg, our administration protocol results in a dose of 166.8 mg human dose [81] that is in a similar range as in a recent clinical study (187.5 mg) on MDMA’s therapeutic effects [25] and can be found in purer MDMA tablets for recreational use [2, 82].

For control animals SAL was used i.p. in equivalent volumes (1 ml/kg). The MDMA-treated and control groups consisted of 11 and 10 animals, respectively, and were randomly assigned to each group. Vehicle-containing Alzet 2001 osmotic minipumps (Durect Corp., CA, USA) were inserted under the skin for all animals. The rats were sacrificed 3 weeks after the injections.

### RNA extraction and sample preparation

Three weeks after MDMA or vehicle injections rats were killed quickly by decapitation. The brains were removed, approximately 2 mm thick coronal sections were cut and the HC, FC and DR regions were dissected according to Paxinos and Watson ([83], dorsal HC: from bregma − 2.5 mm to − 4.5 mm; FC: from bregma + 1.7 to − 0.3 mm; DR: from bregma − 7 mm to − 8 mm, respectively) and stored at − 80 °C. The samples were homogenized with 1 ml TRIzol reagent (Ambion, TX, USA) according to the manufacturer’s instructions. Thus, the homogenized samples were centrifuged at 12000 g at 4 °C for 10 min, the supernatant transferred to a new sterile Eppendorf tube and incubated at room temperature for 5 min. Chloroform in a volume of 200 μl was added; the mixture was vortexed and incubated again at room temperature for 2–3 min. Following centrifugation at 12000 x g at 4 °C for 15 min the upper (clear) aqueous phase was transferred to a new Eppendorf tube and was mixed with 500 μl of isopropanol and incubated for 10 min at room temperature. After centrifuging the samples at 12000 x g at 4 °C for 10 min the supernatant was removed and 1 ml 75% ethanol was added to the precipitation. The samples were again centrifuged at 7500 x g at 4 °C for 5 min, the supernatant was removed, and 1 ml 75% ethanol was added. After centrifuging samples at 7500 x g at 4 °C for 5 min, the ethanol was removed, and the RNA pellets briefly dried. The pellets were dissolved in 20 μl diethylpyrocarbonate treated-dH_2_O (DEPC-dH_2_O) and the samples stored at − 80 °C until further processing. To determine the quality of the samples 1–2 μl were used for optical density (OD, 260/230 and 260/280 ratios) measurements. The OD ratios were determined for all samples and randomly repeated to evaluate the reliability of the measurements (no significant difference was observed, data not shown). Samples with the lowest RNA concentrations were excluded from further analysis and thus both MDMA and control groups consisted of 8 animals. Two-two randomly selected samples were pooled in each treatment group resulting in 4 pooled samples per brain region and per treatment group. These samples (altogether 24 samples) were sent to Service XS (Leiden, Netherlands) for microarray analysis with the Illumina (San Diego, CA, USA) RatRef-12 v1 beadarray expression chip. Upon arrival, samples were once again subjects to a purification process and quality control measurements with Agilent Bioanalyzer and Nanodrop spectrophotometer and one sample from the DR region was excluded from further analysis due to degradation.

### Data analysis

Raw microarray data were processed with beadarray [84], preprocessCore [85] and puma [86] Bioconductor [87] packages for R [88] as described in [89–91]. Briefly, backgroundCorrect method used in the beadarray package was set to “minimum”, and “log = TRUE; n = 10” variables were used for createbeadsummaryData method. The normalization method used was the quantile normalization method in the preprocessCore package. Additionally, pumaComb, pumaDE, and write.rslts functions with default settings were used. Changes were considered statistically significant when the MinPplr was below 0.001. This strict criterion was necessary to reduce the number of false positive results to an acceptable limit.

Heatmap visualization of the differences in gene expression was done using Multiexperiment Viewer Tool [92, 93]. Genes with similar expression patterns are grouped together with hierarchical clustering (Euclidean distance, average linkage) [94].

GSEA was performed using GSEA version 3.1 from the Broad Institute at MIT (http://www.broadinstitute.org/gsea) [95, 96]. Gene sets (GMT format) were obtained from the MSigDB for C5 category (GO gene sets) and in addition, neuronal function related gene sets were selected from the GO homepage (www.geneontology.org; [97]) manually. Gene identifiers used in the array dataset and gene sets were gene symbols. The data set had 22,523 features (Illumina probes), which were collapsed to gene symbols (the median expression value was used for the probe set). In these analyses, the gene sets analyzed were restricted to those sets containing between 15 and 500 genes as recommended [98]. The t-test was used as the metrics for ranking genes and gene set was chosen as the permutation type because of the sample size in the study. One thousand permutations were used to calculate *p*-value with the seed of permutation set to 149. All other basic and advanced fields were set to default. A normalized enrichment score (NES) was calculated for each gene set to represent the degree in which it was enriched in one phenotype. The nominal p-value and the FDR corresponding to each NES were calculated. A NES with a nominal p-value < 0.05, FDR < 0.25 were considered statistically significant. Network visualization and analysis using enrichment results was done using Cytoscape 2.8.3. and its plugin “Enrichment Analyzer” with the following cut-offs: similarity coefficient cut-off 0.1, p-value cut-off 0.05 and FDR cut-off 0.25 [98–100].

### PCR validation

We have validated altogether 19 RNA products from the original pooled samples with real-time PCR on Fluidigm GEx array (San Francisco, CA, USA) using Taqman Gene Expression assays for the appropriate RNAs obtained from Applied Biosystems (Carlsbad, CA, USA) (for the full list of validated genes see Additional file 4: Table S4). Each sample was used in duplo following quality control measurements (altogether three samples were excluded due to degraded or insufficient amount of RNA). The validation experiment was performed by Service XS (Leiden, Netherlands). The Pearson correlation coefficients of the fold change values of the quantile normalized microarray and the housekeeper normalized PCR values were 0.619 and 0.610 for the 200 ng and 500 ng samples, respectively. To provide support to our main conclusions, the array and PCR fold change values of the extensively discussed significant genes included in the validation study (*Gria3, Camk2g, Camk2b, Grin2b and Cnr1)* in the FC and HC are given in Additional file 5: Table S5.

## Additional files

Additional file 1: **Table S1.** contains significantly up- or downregulated genes compared to the control group in the three examined brain region. (XLSX 123 kb)

Additional file 2: **Table S2.** contains individually selected GO terms for GSEA analysis. (XLSX 42 kb)

Additional file 3: **Table S3.** contains the significantly enriched GO terms selected by NES, FDR and nominal *p*-values (nomP) in the examined brain regions. (XLSX 15 kb)

Additional file 4: **Table S4.** contains the genes validated with the Fluidigm GEx PCR array and the used TaqMan assays. (XLSX 18 kb)

Additional file 5: **TableS5.** contains the fold change values of genes validated and extensively discussed in the manuscript measured by Illumina RatRef-12 v1 beadarrays and Fluidigm GEx PCR method after normalization. (XLSX 10 kb)

## Abbreviations

11β-HSD1 (*Hsd11b1*): 11 beta-hydroxysteroid dehydrogenase type 1 (gene)
5-HT: 5-hydroxytryptamine, serotonin
AMPA: α-amino-3-hydroxy-5-methyl-4-isoxazolepropionic acid
AMPA3 (*Gria3*): Glutamate ionotropic receptor AMPA type subunit 3 (gene)
ATP2B1 (*Atp2b1*): ATPase, Ca++ transporting, plasma membrane 1 (gene)
ATP2B3 (*Atp2b3*): ATPase, Ca++ transporting, plasma membrane 3 (gene)
BDNF (*Bdnf*): Brain-derived neurotrophic factor (gene)
Camk1g (*Camk1g*): Calcium/calmodulin-dependent protein kinase I gamma subunit (gene)
Camk2g/2b (*Camk2g/2b*): Calcium/calmodulin-dependent protein kinase II gamma/beta subunits (genes)
*Camk2n2*: Calcium/calmodulin-dependent protein kinase II inhibitor 2 gene
CaMKII: Calcium/calmodulin dependent protein kinase II
CB1 (*Cnr1*): Cannabinoid receptor type 1 (gene)
CNS: Central nervous system
CYP2D1: Cytochrome P450 2D1
CYP2D6: Cytochrome P450 2D6
DA: Dark Agouti
*Dao1*: D-amino oxidase gene
DEPC-dH_2_O: Diethylpyrocarbonate treated-dH_2_O
DR: Dorsal raphe
EphA4 (*Epha4*, *LOC316539*): Ephrin-type A receptor 4 (gene)
EphA5 (*Epha5*): Ephrin-type A receptor 5 (gene)
EphA6 (*Epha6*): Ephrin-type A receptor 6 (gene)
FC: Frontal cortex
FDR: False discovery rate
fMRI: Functional magnetic resonance imaging
GABA-A: Gamma-aminobutyric acid A receptor
*Gabre*: Gamma-aminobutyric acid A receptor, epsilon subunit gene
GO: Gene ontology
GSEA: Gene set enrichment analysis
HC: Hippocampus
*Hsf2*: Heat shock factor 2 gene
HSP90α (*Hspca*): Alpha subunit of the heat shock protein 1 (gene)
HSPs: Heat-shock proteins
i.p.: Intraperitoneally
*Kalrn*: Kalirin gene
KO: Knockout
Kv3.1b (*Kcnc2*): Potassium voltage gated channel, Shaw-related subfamily, member 2 (gene)
Kv4.2 (*Kcnd2*): Potassium voltage-gated channel, Shal-related family, member 2 (gene)
LTP: Long-term potentiation
MDMA: 3,4-methylenedioxymethamphetamine
MinPplr: Minimum probability of positive log ratio
mRNA: Messenger RNA
MsigDB: Molecular signatures database
NES: Normalized enrichment score
NMDA: N-methyl-D-aspartate
NMDA2A/GRIN2A (*Grin2a*): N-methyl D-aspartate receptor subtype 2A/Glutamate receptor, ionotropic subtype 2A (gene)
NMDA2B/GRIN2B (*Grin2b*): N-methyl D-aspartate receptor subtype 2B/Glutamate receptor, ionotropic subtype 2B (gene)
OD: Optical density
PCR: Polymerase chain reaction
PFC: Prefrontal cortical regions
PTSD: Post-traumatic stress disorder
RhoGEFs: Guanine nucleotide exchange factors that activate Rho GTPases
RhoGTPase: Rho family of GTP-binding protein regulators
RNA: Ribonucleic acid
SAL: Saline
SEM: Standard error of the mean
*Slc1a3*: Solute carrier family 1 (glial high affinity glutamate transporter), member 3 gene
*Slc5a3*: Solute carrier family 5 (inositol transporters), member 3 gene
*Slc6a5*: Solute carrier family 6 (neurotransmitter transporter, glycine), member 5 gene
